# What is the best method for debonding metallic brackets from the patient’s perspective?

**DOI:** 10.1186/s40510-015-0088-7

**Published:** 2015-06-17

**Authors:** Matheus Melo Pithon, Daniel Santos Fonseca Figueiredo, Dauro Douglas Oliveira, Raildo da Silva Coqueiro

**Affiliations:** Av. Otavio Santos, 395 sala 705, Centro Odontomédico Dr. Altamirando da Costa Lima, Vitoria da Conquista, Bahia, 45020750 Brazil; Southwest Bahia State University UESB, Jequié, Bahia Brazil; Pontifical Catholic University of Minas Gerais, Belo Horizonte, Brazil

**Keywords:** Pain, Orthodontics brackets, Orthodontic treatment

## Abstract

**Background:**

The aim of this clinical investigation was to compare the level of discomfort reported by patients during the removal of orthodontic metallic brackets performed with four different debonding instruments.

**Methods:**

The sample examined in this split-mouth study comprised a total of 70 patients (840 teeth). Four different methods of bracket removal were used: lift-off debonding instrument (LODI), straight cutter plier (SC), how plier (HP), and bracket removal plier (BRP). Prior to debonding with all experimental methods, the archwire was removed. Before appliance removal, each patient was instructed about the study objectives. It was explained that at the end of debonding in each quadrant, it would be necessary to assess the discomfort of the procedure using a visual analog scale (VAS). This scale was composed of a millimeter ruler scoring from 0 to 10, in which 0 = a lot of pain, 5 = moderate pain, and 10 = painless. The level of significance was predetermined at 5 % (*p* = 0.05), and the data were analyzed using the BioEstat 5.0 software (BioEstat, Belém, Brazil).

**Results:**

The pain scores with SC were significantly higher than in all other methods. There were no significant differences between HP and BRP pain scores, and the LODI group showed the lowest pain scores. Statistically, significant differences were observed in the ARI between the four debonding methods.

**Limitations:**

The biggest limitation of this study is that each tooth was not assessed individually.

**Conclusions:**

Patients reported lower levels of pain and discomfort when metallic brackets were removed with the LODI. The use of a straight cutter plier caused the highest pain and discomfort scores during debonding.

## Background

Despite all recent developments in dentistry, patient’s complaints of pain or discomfort are commonly registered after different types of dental treatments and orthodontic therapy is not an exception [1, 2]. The existing literature shows that procedures such as the use of elastic separators, archwire placement and activations, as well as the application of orthopedic forces may cause pain in orthodontic patients [3–5]. It is also known that the possibility of experiencing pain or discomfort may negatively influence the willingness of patients to undergo orthodontic treatment [6–9].

Orthodontic patients may experience pain not only during the phase of active treatment but also during the removal of fixed appliances [10]. Various methods to debond metallic and ceramic brackets have been described in the literature, including the use of special debonding pliers [8], ultrasound [9, 10] or laser application [11, 12], electrothermic debonding [13–15], special instruments [8, 16], and the use of bonding materials presenting thermoexpandable microcapsules to facilitate debonding [17]. Despite the several techniques described, few authors have been concerned about understanding the discomfort the different debonding methods cause to orthodontic patients [10, 12]. Undoubtedly, the ideal debonding method should be harmless to the enamel and painless to the patients [18–20]. However, researchers have been more focused on studying pain during orthodontic treatment and the technical details of debonding rather than evaluating ways to minimize patients’ discomfort during debonding [21, 22].

In an attempt to fill this gap in the literature, the aim of this clinical investigation was to compare the level of discomfort reported by patients during the removal of orthodontic metallic brackets performed with four different debonding instruments and to verify the integrity of the enamel after debonding.

## Methods

Before data collection began, the project was sent for approval by the research ethics committee of Southeast Bahia State University, and it received a favorable report (number 405.944). The sample examined in this split-mouth study was collected from a single private practice and included patients who had fixed orthodontic appliances removed in both arches. The inclusion criteria were the presence of an Angle class I malocclusion and permanent teeth, except the third molars. Conversely, patients requiring extractions or presenting teeth with restorations on the buccal surface were excluded from the study. The sample comprised a total of 70 female patients (840 teeth). The mean age at the time of debonding was 31 years and 10 months, ranging from 14 years and 3 months to 45 years and 11 months. All patients or their legal guardians signed an informed consent prior to initiating their treatment.

All fixed appliances were bonded and removed by the same orthodontist, and the same brand and model of stainless steel brackets were used on all patients (Standard Edgewise 0.022 × 0.030 in.) (Morelli, Sorocaba, Brazil). Prior to bonding, the buccal surface of all teeth was pumiced with a mixture of pumice and water on slow speed, enamel was conditioned with a self-etching primer (SEP 3M/Unitek, Monrovia, CA, USA), and the same resin composite (Transbond XT 3M/Unitek, Monrovia, CA, USA) was used in all patients following the manufacturer’s instructions.

Before appliance removal, each patient was instructed about the study objectives. It was explained that at the end of debonding in each quadrant, it would be necessary to assess the discomfort of the procedure using a visual analog scale (VAS). This scale was composed of a millimeter ruler scoring from 0 to 10, in which 0 = a lot of pain, 5 = moderate pain, and 10 = painless. Prior to debonding, the order and the debonding method for each quadrant were randomly selected. The teeth evaluated in this study were canine and premolars because their brackets were exactly the same.

**Fig. 1:**
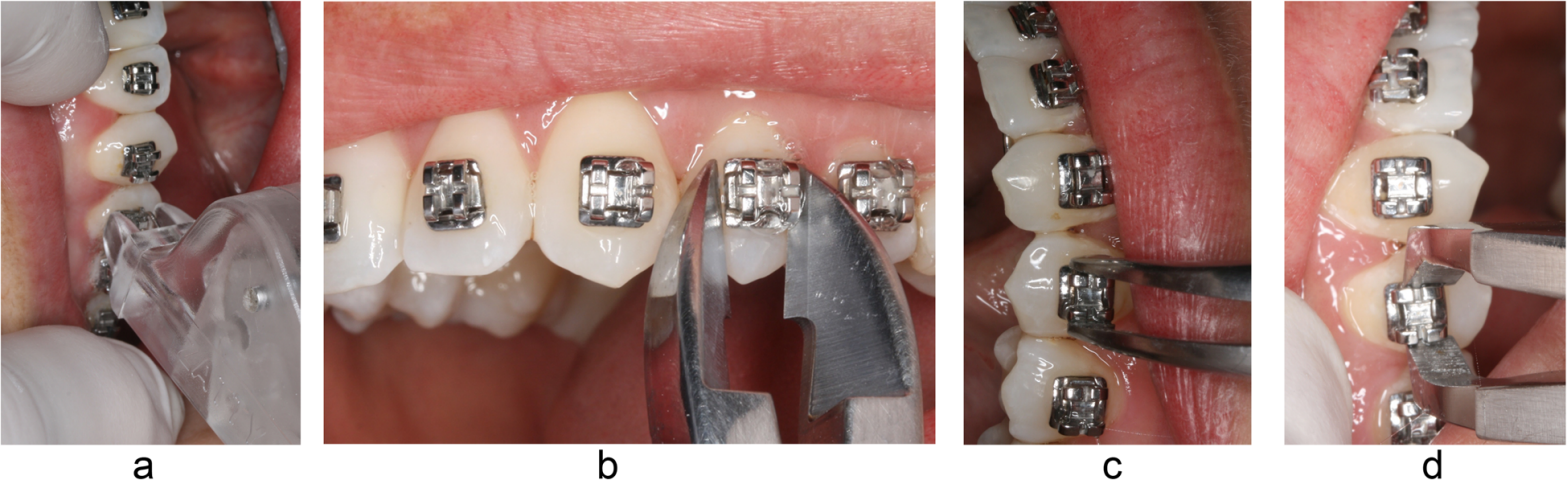
Debonding methods used. **a** Lift-off debonding instrument (LODI). **b** Straight cutter (SC). **c** How plier (HP). **d** Bracket removal plier (BRP)

Four different methods of bracket removal were used (Fig. 1). The first method examined was the lift-off debonding instrument (LODI) (Zatt, São Paulo, Brazil), in which a pull wire was engaged under the bracket wing producing a pulling force after a light squeeze of the handles while both plastic rests were placed on the tooth surface. The second debonding method tested was a straight cutter plier (SC) that was used to grab the bracket wings, applying pressure to the bracket base mesially and distally. The third method was the use of a how plier (HP) to press both mesial and distal wings, deforming the bracket base. The last method tested was a bracket removal plier (BRP), as shown in Fig. 1d. Prior to debonding with all experimental methods, the archwire was removed.

**Fig. 2:**
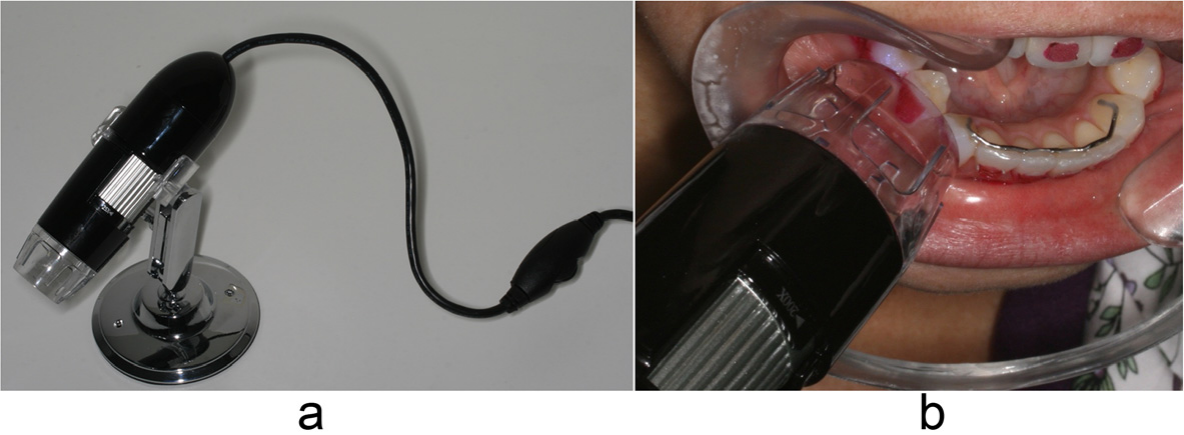
**a** Portable digital microscope used to determine the ARI. **b** Microscope being used

**Fig. 3:**
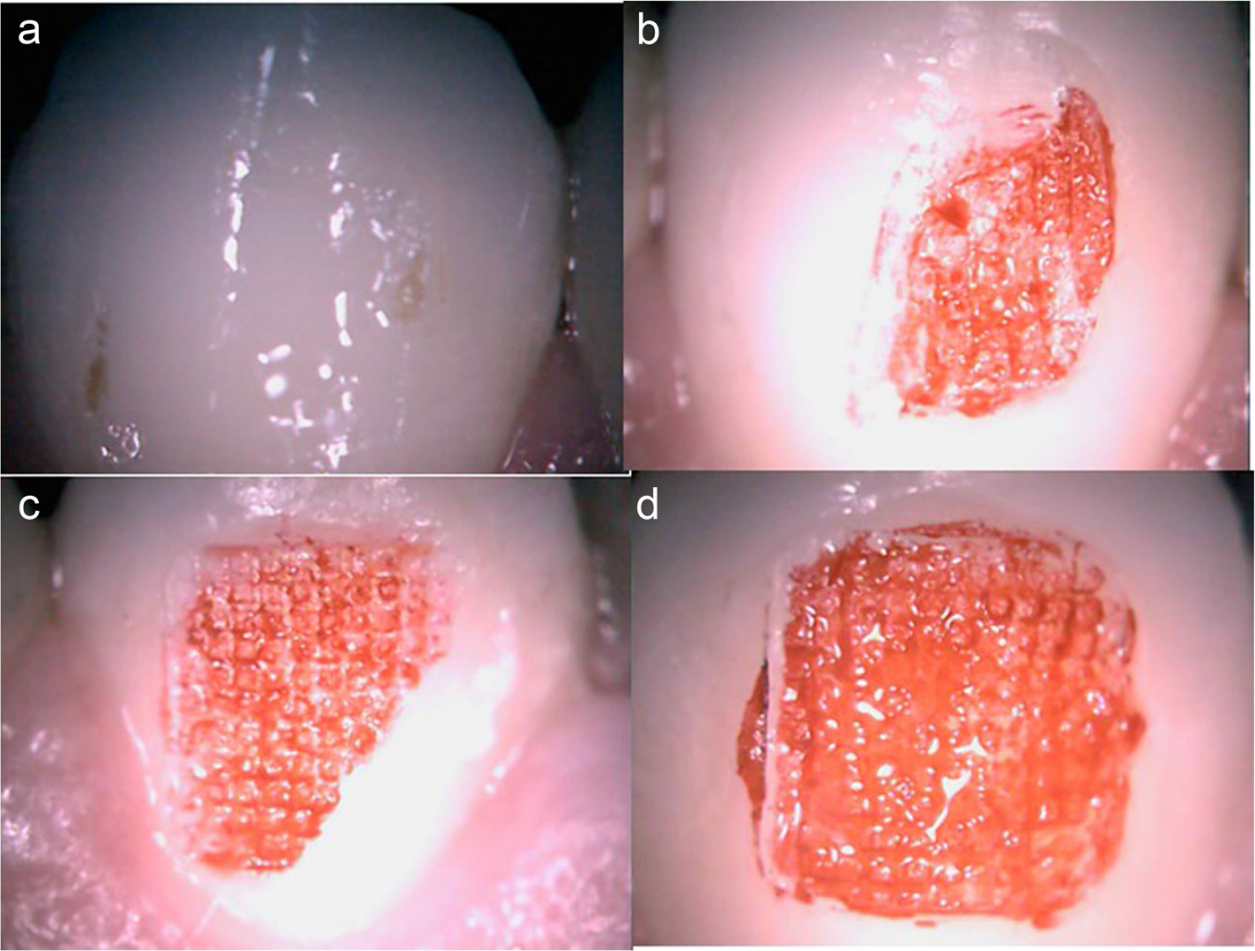
Images obtained with the patient directly into portable microscope. **a** IRA = 0. **b** IRA = 1. **c** IRA = 2. **d** IRA = 3

The amount of composite remaining on the enamel surface was examined immediately after bracket removal. The patients were instructed to rinse with a solution containing fuccina (Biodinâmica Química e Farmacêutica LTDA, Ibiporã, Brazil) for better identification of residual composite adhered to the tooth. A portable electron microscope (Vehs, Hong Kong, China) was used to evaluate the adhesive remnant index (ARI) (Fig. 2a, b) [23]. The scale had scores ranging from 0 to 3, where 0 = no adhesive remaining; 1 = less than half of adhesive remaining; 2 = more than half of adhesive remaining; and 3 = all adhesive remaining (Fig. 3). All values were assigned by a single orthodontist.

### Statistical analyses

For descriptive analysis of pain scores and ARI values, mean and standard deviations were calculated. The differences registered for all debonding methods were compared with the Friedman test followed by the Wilcoxon test to compare pairs. The level of significance was predetermined at 5 % (*p* = 0.05), and the data were analyzed using the BioEstat 5.0 software (BioEstat, Belém, Brazil).

## Results

**Fig. 4:**
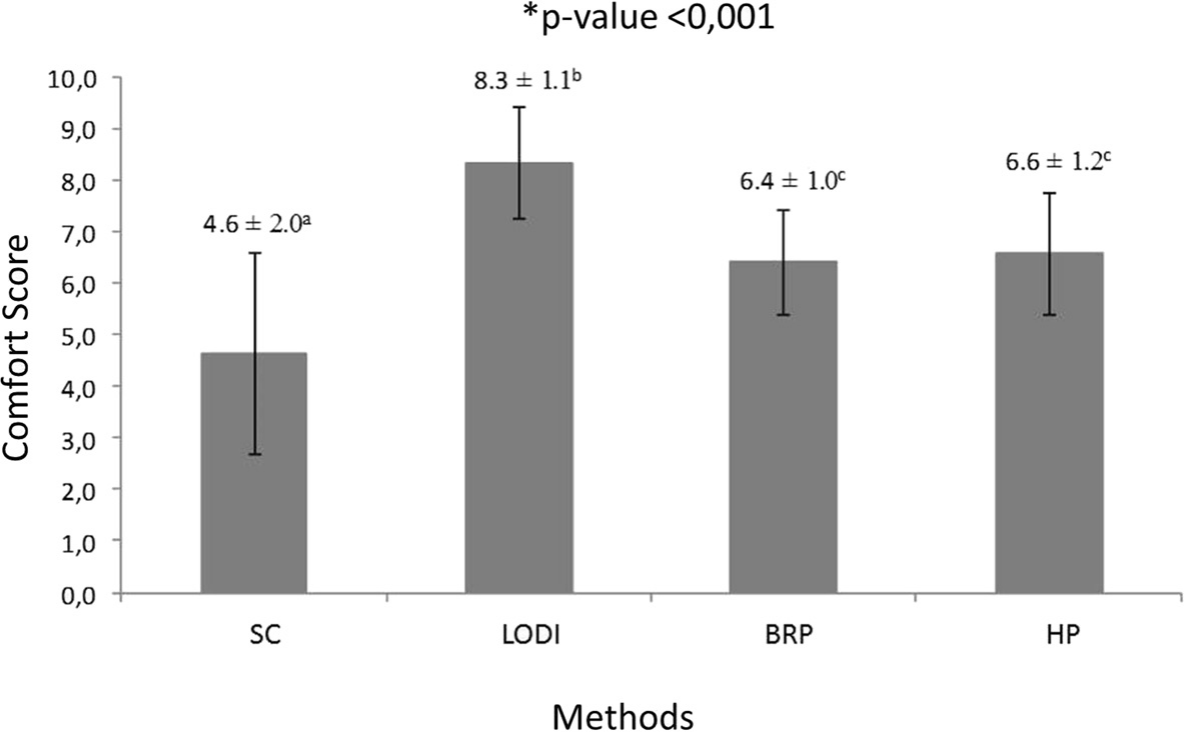
Mean ± standard deviation perception of discomfort scores during bracket debonding, according to the different methods used. *Statistical difference between the methods (Friedman test). Different letters (*superscript a*, *b*, *c*) represent statistical difference (Wilcoxon test)

Figure 4 shows the perception of discomfort reported by the participants according to the debonding method used. The Friedman test indicated that pain scores were statistically significantly different depending on the debonding method. Comparisons between pairs by the Wilcoxon test showed that pain scores with SC were significantly higher than in all other methods. There were no significant differences between HP and BRP pain scores, and the LODI group showed the lowest pain scores.

**Fig. 5:**
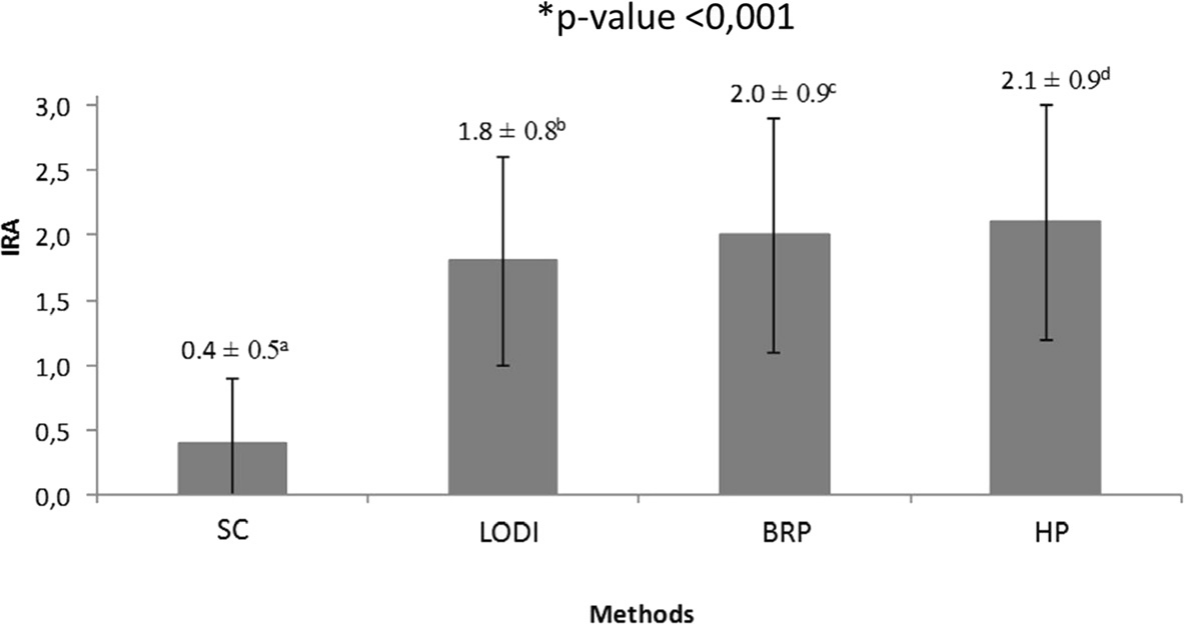
Mean ± standard deviation ARI scores registered with the four debonding methods tested. *Statistical difference between the methods (Friedman test). Different letters (*superscript a*, *b*, *c*) represent statistical difference (Wilcoxon test)

Statistically significant differences were observed in the ARI between the four debonding methods (Fig. 5). Comparisons between pairs by the Wilcoxon test showed that the methods of debonding brackets that promoted the greatest ARI were: HP > BRP > LODI > SC.

## Discussion

The search for ways to reduce patient’s pain and discomfort during different dental procedures has been a concern in contemporary dentistry [21, 22]. The same concern exists in orthodontics [4], however, relatively few publications on this topic have been found in the literature, especially when compared to other areas of orthodontic research [4]. For example, despite the various methods of debonding described in the literature [24–27], few have been studied in relation to pain or discomfort they may cause to the orthodontic patients. Thus, the purpose of the present paper was to evaluate four methods commonly used to debond metallic brackets, determining possible differences in patient’s discomfort and their amount of adhesive remaining on the enamel surface after debonding.

The results of this study showed significant differences on the patient’s discomfort between the four debonding methods. In general, the use of LODI caused lower levels of pain or discomfort to remove the brackets when compared to the other methods. Furthermore, the use of the SC was the method that presented the highest discomfort for the patients. This finding corroborates with previous clinical reports [28, 29], as well as the study of Normando et al. [12], which also compared quantitatively the discomfort caused by LODI and SC. However, that study [12] was limited to these two methods and did not evaluate other methods widely used by orthodontists, such as HP and BRP. In our study, HP and BRP showed similar scores of patient discomfort that reached intermediate levels between SC and LODI mean discomfort values.

The reasons that justify the different results found between the methods studied are unknown. However, the direction of the debonding force may influence the degree of discomfort during the removal of metallic brackets [10].

According to Williams and Bishara [10], the direction of debonding force application and the mobility of the tooth along with gender differences are seen to be factors influencing the discomfort threshold. Patients can withstand intrusive forces significantly more than forces applied in a mesial, distal, facial, lingual, or an extrusive direction [10]. Unfortunately, we have no way to standardize the direction as each instrument for removing presents a different mode of operation. However, during removal, the manufacturer’s recommendations regarding the use of these instruments were followed. Therefore, the similar force systems created by the different instruments during debonding may provide an explanation for these findings. BRP, SC, and HP exert forces of similar magnitude, but in opposite direction. They cancel each other requiring greater amounts of force applied by the operator, which could lead to greater patient discomfort. Conversely, the LODI may require lower and more constant force levels, which may be linked to a lower score of pain or discomfort [12] Williams and Bishara [10] suggested that approximately 1000 g should be considered as an “appropriate force” limit to be directly applied to a tooth.

The discomfort felt in the premolars and canines were recorded in this study. This was an attempt to better standardize the sample. Primarily, because the brackets used for both teeth were identical. Secondly, because the type of tooth appears to influence on the threshold of pain, since the biggest complaints have been previously reported during incisor debonding [12]. This may be explained because the tactile sensory thresholds of normal people were about 1 g in the anterior portion of the dental arch and gradually increased toward the posterior segments of the arch, ranging from 5 to 10 gm [30]. Conversely, patient’s gender appear to have little influence on the threshold of discomfort [10]; thus, we decided not to distinguish the groups by gender.

The four methods of debonding were also assessed for their damaging potential to the enamel surface. A crucial point for this evaluation is the line of adhesive fracture during bracket debonding. According to Artun and Bergland [23], the enamel would be protected if the adhesive line of fracture was located exclusively within the adhesive layer; thus, a thin layer of adhesive would remain attached to the enamel after debonding, covering 100 % of the bracket base’s previous location, instead of having a line of fracture at the enamel-adhesive interface) [15]. Therefore, the ARI is an important indicator when assessing the integrity of the enamel surface after bracket debonding.

A portable electron microscope was used to evaluate the ARI, which due to its reduced dimension could be introduced directly into the oral cavity. After debonding and prior to microscopic evaluation, the enamel was stained with fuccina to facilitate the composite visualization and quantification.

The results of this study showed significant ARI differences among the four debonding methods tested. Despite these statistical differences, HP, LODI, and BRP showed clinically similar results, showing on average, approximately half of the enamel surface with remaining adhesive (Fig. 5). However, the ARI for SC was noticeably smaller than those registered for the other groups (Fig. 5), which seems to indicate greater potential for enamel damage. SC generates forces directly to the adhesive layer and below the bracket base, immediately transferred to the underlying enamel [28], thereby presenting higher risks to injure the enamel [14].

Therefore, when compared to the other methods tested, the use of SC seems to be far from the ideal debonding method, since it has greater potential to cause enamel damage which caused greater patient discomfort. Furthermore, if bracket rebonding is required, the use of SC may also not be considered the method of choice for removal of the brackets since another study [14, 15] reported that the majority of the brackets debonded with SC showed significant structural deformations at the base and/or at the slot. Conversely, LODI showed the highest patient acceptability. Moreover, previous reports showed that all brackets debonded with the LODI remained structurally intact afterward [14, 15], indicating that this method may also be the most recommended when bracket rebonding is necessary.

## Conclusions

According to our findings, it can be concluded that:

- Patients reported lower levels of pain and discomfort when metallic brackets were removed with the lift-off debonding instrument.
- How plier and bracket removal plier generated intermediate and similar levels of patient discomfort.
- The use of a straight cutter plier caused the highest pain and discomfort scores during debonding.
- The ARI scored with all four debonding methods were not significantly different.
